# Vitamin D Supply of Multivitamins Commercialized Online by Amazon in Western and Southern Europe: A Labeling Analysis

**DOI:** 10.3390/nu15020326

**Published:** 2023-01-09

**Authors:** Lisa Ponti, Luca Gabutti, Pietro B. Faré, Simone Janett, Mario G. Bianchetti, Peter J. Schulz, Sebastiano A. G. Lava, Carlo Agostoni, Gregorio P. Milani

**Affiliations:** 1 Department of Internal Medicine, Clinical Research Unit, Regional Hospital of Bellinzona and Valli, Ente Ospedaliero Cantonale, 6500 Bellinzona, Switzerland; 2 Family Medicine, Faculty of Biomedical Sciences, Università della Svizzera Italiana, 6900 Lugano, Switzerland; 3 Faculty of Biomedical Sciences, Università della Svizzera Italiana, 6900 Lugano, Switzerland; 4 Division of Infectious Diseases, Department of Medicine, Ente Ospedaliero Cantonale, 6900 Lugano, Switzerland; 5 Department of Pulmonology, University Hospital Zurich, 8091 Zurich, Switzerland; 6 Department of Communication and Media, Ewha Womans University, Seoul 03760, Republic of Korea; 7 Pediatric Cardiology Unit, Department of Pediatrics, Centre Hospitalier Universitaire Vaudois, University of Lausanne, 1011 Lausanne, Switzerland; 8 Heart Failure and Transplantation, Department of Paediatric Cardiology, Great Ormond Street Hospital, London WC1N 3JH, UK; 9 Pediatric Unit, Fondazione IRCCS Ca’ Granda Ospedale Maggiore Policlinico, 20122 Milan, Italy; 10 Department of Clinical Sciences and Community Health, Università degli Studi di Milano, 20122 Milan, Italy

**Keywords:** Amazon, multivitamins, online shopping, vitamin D, overdose

## Abstract

Multivitamins are commonly used by the general population, often without medical prescription. The purpose of this report is to inform on the daily vitamin D supply provided by multivitamins containing vitamin D that are commercialized online by Amazon in Western and Southern Europe. We surveyed multivitamins aimed at adults using the following marketplaces: amazon.es^®^, amazon.de^®^, amazon.it^®^, and amazon.fr^®^. We identified 199 vitamin D_3_-containing multivitamins sold by Amazon marketplaces: 77 from amazon.es^®^, 73 from amazon.de^®^, 33 from amazon.it^®^, and 16 from amazon.fr^®^. No multivitamin contained vitamin D_2_. The daily vitamin D_3_ supply ranged from 16 to 2000 IU: it was less than 400 IU daily in 108 (54%), 400–800 IU daily in 53 (27%), and more than 800 IU daily in the remaining 38 (19%) products. The vitamin D_3_ supply of products sold by amazon.it^®^ was on average higher (*p* < 0.05) than that of products sold by amazon.de^®^, amazon.fr^®^, and amazon.es^®^. In conclusion, the vitamin D supply of multivitamins sold by Amazon may be insufficient, marginally sufficient, or adequate for subjects at high risk of hypovitaminosis D such as subjects 65 years or more of age, pregnant (or lactating) women, or patients on drug treatment or with an underlying disease, where a vitamin D supplementation is advocated.

## 1. Introduction

An increasing number of individuals take multivitamin (and multimineral) supplements to maintain good health and to be protected from different diseases including cardiovascular disease, cancer, and decline in cognitive function [1]. However, now that diets are more assorted, supplemented, and fortified, diseases of overt vitamin deficiency are rare, and the most commonly occurring diseases have a multifactorial cause. Hence, it may be unlikely for a supplement to confer a health benefit [1]. This impression is supported by studies and meta-analyses demonstrating that vitamin and mineral supplements do not provide benefits to individuals in the general population [1,2,3]. Nevertheless, these products, which can be purchased without medical prescription, are very commonly used by the general population [4] and are often recommended in naturopathic medicine [5].

Hypovitaminosis D has long been known for its role in calcium homeostasis and skeletal health [6]. In more recent years, vitamin D has been postulated to play a role in various disorders including cancer, cardiovascular disease, diabetes, psychiatric illness, and infection as well as allergic, immunologic, and inflammatory disorders. Vitamin D appears to affect the production of pro-inflammatory cytokines as well as the proliferation of pro-inflammatory cells. In this capacity, it might play a role in the modulation of both the immune and inflammatory responses, both of which are of primary importance in the development of inflammatory diseases [6].

Indeed, 25-hydroxyvitamin D is the main circulating metabolite of vitamin D, and experts currently agree that its total concentration is the best easily available measure for vitamin D status [6]. Although the optimal 25-hydroxyvitamin D blood level is controversial, most authorities currently favor maintaining a concentration between 50 and 100 nmol/L for both skeletal and extra-skeletal health [6]. Many reports point out that in adults, a 25-hydroxyvitamin level below 50 nmol/L is highly prevalent worldwide. An age of 65 years or more, increased skin pigmentation, reduced effective sun exposure due to protective clothing or use of sunscreens, excessive body weight, medication that accelerates the metabolism of vitamin D, and malabsorption (including inflammatory bowel disease and celiac disease) are the main risk factors for hypovitaminosis D [6]. Most authorities currently recommend a supplementation with vitamin D 800–1000 IU daily to subjects 65 or more years of age [6,7,8,9]. Many authorities also support vitamin D supplementation (usually 600 IU daily) during pregnancy and lactation [6,7,9]. Although the potential mutual relationship between excessive body weight and low levels of vitamin D is still a matter of debate, vitamin D supplementation is also recommended for subjects with extremely increased body fat [10]. Finally, vitamin D supplementation is advised in patients of any age taking drugs that interfere with the vitamin D metabolism (e.g., antiepileptics, antivirals, corticosteroids) or with intestinal malabsorption (e.g., Crohn’s disease, ulcerative colitis, celiac diseases, and cystic fibrosis), diabetes mellitus, chronic kidney or liver disease, reduced bone density, and after weight-loss surgery [6,7].

In the past years, online shopping of both non-prescription (and prescription) medicines and multivitamins has massively increased [11,12]. It is unclear, however, whether the daily vitamin D supply of multivitamins commercialized online provides the recommended supplementation of vitamin D or lends the potential for an increased occurrence of vitamin D toxicity. Indeed, excessive supplementation of vitamin D might be associated with side effects [13]. The purpose of this labeling analysis is to inform on the daily vitamin D supply provided by multivitamins containing vitamin D, which are commercialized online by Amazon marketplaces in Western and Southern Europe.

## 2. Materials and Methods

### 2.1. Search Strategy

We surveyed multivitamin products for adult use on the following Amazon marketplaces: amazon.es^®^, amazon.de^®^, amazon.it^®^, and amazon.fr^®^. The search through Amazon websites was conducted in January 2022. Two researchers independently conducted the research using the Amazon’s search bar of the four marketplaces. The following search terms were used: “multivitamins”, “multivitamins with vitamin D”, and “multiple vitamin and mineral combination”. The content of each product resulted from the search was screened to ascertain the content of vitamin D. A product was considered a multivitamin supplement if the packaging included the term multivitamin (or a derivative of this) or if two or more vitamins were named in addition to vitamin D. After the selection of the eligible products, those without any information on the vitamin D amount were excluded. Multivitamins explicitly commercialized for newborns, infants, and children were also excluded. To determine the daily vitamin D supply provided by a multivitamin product, when a dose range was recommended (e.g., 1 to 3 pills per day), the highest amount was recorded. Data regarding the name of other vitamins contained in the preparation were also recorded. At the end of the product selection, an author recorded the data into a predefined database and another author verified data entry accuracy. Controversies in product selection and data extraction were solved by consensus.

### 2.2. Analysis

The vitamin D-containing multivitamin products were subdivided into three groups: those with a daily supply of less than 400 IU, those with a supply ranging between 400 and 800 IU, and those with a supply of more than 800 IU. The study results are presented either as absolute frequency and percentage for categorical variables, or as median and interquartile range for continuous variables. For inferential statistics, the Kruskal–Wallis test with the post-hoc Tukey procedure was employed [14]. A two-tailed significance level of 0.05 or less was used. Statistics was performed using the open source statistical language R version 3.5.3 (R Core Team, Vienna, Austria).

## 3. Results

We identified a total of 199 vitamin D_3_ (cholecalciferol)-containing multivitamin products sold by Amazon marketplaces: 77 from amazon.es^®^ (Supplementary Materials Table S1), 73 from amazon.de^®^ (Supplementary Materials Table S2), 33 from amazon.it^®^ (Supplementary Materials Table S3), and 16 from amazon.fr^®^ (Supplementary Materials Table S4). No multivitamin product contained vitamin D_2_ (ergocalciferol).

**Figure 1:**
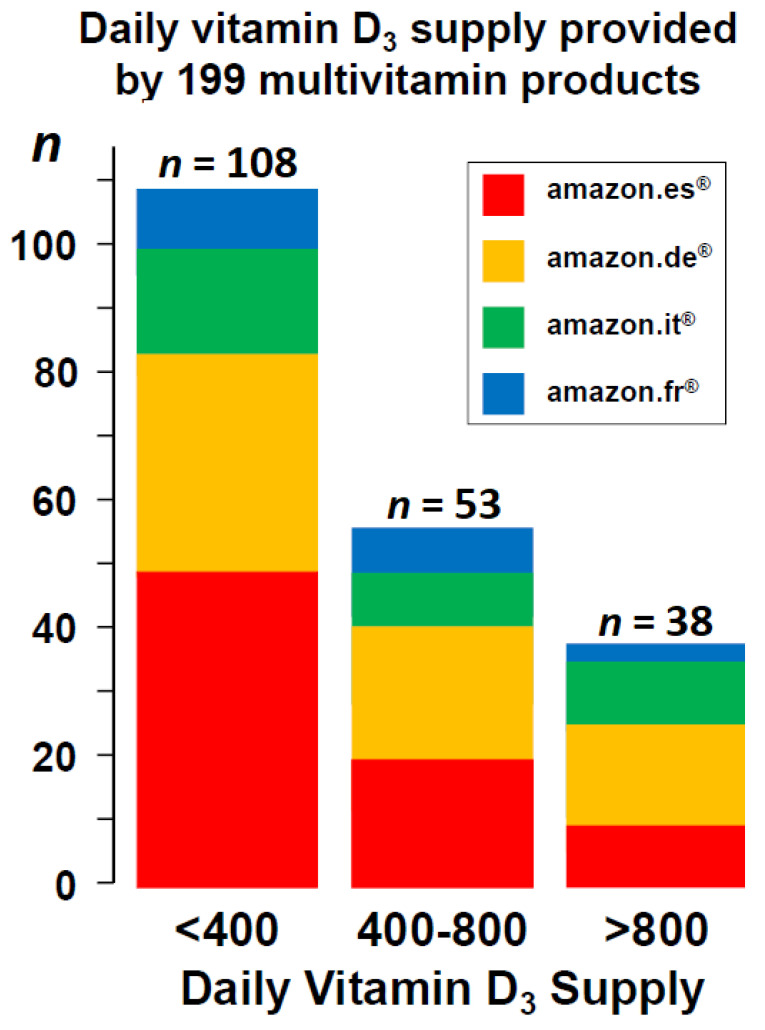
Daily vitamin D_3_ supply provided by 199 multivitamin products.

The daily vitamin D_3_ dose supply ranged from 16 to 2000 IU. It was less than 400 IU daily in 108 (54%), 400–800 IU daily in 53 (27%), and more than 800 IU daily in the remaining 38 (19%) products (Figure 1 and Table 1).

The vitamin D_3_ supply of multivitamin products sold by amazon.it^®^ was significantly (*p* < 0.05) higher (600 (200–1000) IU daily) than that of products sold by amazon.de^®^ (336 (200–800) IU daily), amazon.fr^®^ (250 (200–500)), and amazon.es^®^ (200 (190–440) IU daily). The number of vitamins other than vitamin D_3_ included in each product is given in Table 2. Approximately 70% of the products contained 11 to 13 vitamins other than vitamin D_3_.

The list of vitamins other than vitamin D contained in each individual product is detailed in Supplementary Materials Tables S1–S4. Vitamin C, vitamin E, vitamin B_1_, vitamin B_6_, and vitamin B_12_ were contained in 90% or more of the products (Table 2).

## 4. Discussion

Multivitamin (and multimineral) supplements became available in the early 1940s [15]. Unfortunately, they do not benefit from standardized scientific, regulatory, or marketplace definitions yet [15,16]. In the United States, these supplements are taken by at least one-third of adults and one-quarter of adolescents [15,17]. The use of dietary supplements is currently increasing also in many European countries [18,19,20]. Recent data also suggest that the use of supplements is particularly elevated in families with a high socioeconomic status [21]. A preliminary analysis of Google Trend data by the authors of this paper shows that the number of searches by consumers on multivitamin products in Italy, Switzerland, Germany, France, the United Kingdom, and the United States has tripled over the past 10 years. Furthermore, supplements are especially sought during the winter season to reduce respiratory tract infections [22]. During the coronavirus disease 2019 pandemic, the risk of hypovitaminosis has been claimed, and many have turned to taking multivitamin supplements in an attempt to prevent or even combat that condition [23,24,25,26]. Finally, the potential of vitamin D in Long COVID modulation has also been proposed [27].

Rickets was clearly recognized by F. Glisson in England and D. Whistler in the Netherlands as early as the seventeenth century [6]. By the turn of the twentieth century, there was an animated debate over whether rickets was the result of an environmental factor or the lack of a dietary factor. Based upon research on the geographical distribution of rickets, T. A. Palm advocated that rickets could be prevented or cured by sunshine [6,28]. This hypothesis was further supported by E. Bucholtz, A. F. Hess, K. Huldshinsky, and J. Raczynski, who conducted investigations in which children with rickets were cured after exposure to sunlight. On the other hand, E. V. McCollum observed [6,28] the anti-rickets properties of cod liver oil (and the term “bottled sunshine” was coined). The dilemma about why both sun exposure and cod liver oil cure rickets was subsequently resolved by studies, which demonstrated the dual role of nutrition and sun exposure in the management of rickets. Further experiments found that the irradiation of foods such as plant oils or yeasts increased their anti-rickets properties. A. Windaus, who was awarded the Nobel Prize in 1928, elucidated the chemical structure of vitamin D and demonstrated, among others, that it exists in two main forms: vitamin D_2_ (ergocalciferol), which is plant-derived; and vitamin D_3_ (cholecalciferol), which is animal-derived and synthetized in the skin [6,28].

Studies recently conducted in Southern and Western Europe suggest that the prevalence of vitamin D < 50 nmol/L largely varies among countries [29,30,31,32]. A study conducted in rural areas of Italy found that approximately half of the 3448 included subjects showed vitamin D levels < 50 nmol/L [30]. A study including ~7000 German adults found that 60% of participants had vitamin D levels < 50 nmol/L [31]. Finally, a French study involving ~900 healthy adults detected vitamin D levels below 50 nmol/L in 34% of the participants [32].

The present analysis addressed the daily vitamin D supply provided by almost 200 vitamin D-containing multivitamins, which are commercialized by amazon.es^®^, amazon.de^®^, amazon.it^®^, and amazon.fr^®^. The supply is mostly less than 400 IU daily (>50% of products). The remaining vitamin D-containing multivitamins supply between 400 and 800 IU daily (approximately one-quarter of products) or more than 800 IU daily (approximately one-fifth of the products). Accordingly, the vitamin D supply of multivitamin preparations may be insufficient, marginally sufficient, or adequate for subjects 65 years or more of age, for pregnant (or lactating) females, or for patients on a drug treatment or with an underlying disease, for which a vitamin D supplementation is currently advocated. On the other hand, since none of the vitamin D-containing multivitamins provide more than 2000 IU vitamin D daily, a further supplementation with approximately 1000 IU daily is likely non-toxic considering that authorities such as the European Food Safety Authority and the Institute of Medicine deem an intake of up to 4000 IU daily as the maximum safe dose for adults [6].

Vitamin D intoxication from a dietary source is likely a rare (but potentially threatening) event whose frequency is unclear, because no studies have focused on this question [33,34,35]. Various causes of vitamin D toxicity have been documented over the last years [26,33,34]. Reports describe cases of accidental or intentional intake of excessive vitamin D caused by a variety of circumstances such as misinterpretation of prescriptions or inappropriate prescription of excessive vitamin D doses. In our opinion, high doses of vitamin D are not rarely prescribed considering that a long-term supplementation with vitamin D in the range of 10,000 to 25,000 IU daily has been considered safe [33,34]. In both Europe and the United States, finally, intoxication has also been reported after manufacturing errors of vitamin D formulations containing considerably higher amounts than claimed on the label [33,34,35]. Excessive exposure to sunlight does not result in vitamin D intoxication because both pre-vitamin D and vitamin D are photolyzed to non-calcemic photoproducts [34,35,36,37].

Beyond sunlight exposure, skin phototype, and body fat, further factors can modulate vitamin D status and its health-associated effects [38]. A recent study showed that adherence to a Mediterranean diet is linked to higher levels of circulating vitamin D [39]. Sex difference is a further potential factor associated with vitamin D levels, with females showing lower vitamin D levels than males, irrespective of the body mass index [40]. Finally, independently from vitamin D levels, their effects on human health might vary according to seasonality [41,42]. The complex relationship between this vitamin and human health highlights the importance of assessing vitamin D intake for self-care.

According to regulatory agencies, compounded pills should contain between 90% and 110% of the active ingredient reported on the label. Previous investigations conducted in the USA found that only one-third of vitamin D pills met such criteria. An elegant investigation by LeBlanc et al. studied the cholecalciferol content of five commercially available, over-the-counter pills. The authors found that the vitamin content of pills ranged from 9% to 146% of the stated dose [43]. These findings, together with the results of this analysis, suggest that authorities should regulate and producers should provide well-defined indications on the amount of vitamin D to be contained in multivitamins. This might help to avoid confusion among both consumers and health professionals and consequent errors in vitamin D intake.

This cross-sectional study has some limitations. The present labeling analysis evaluated the vitamin D supply of approximately 200 multivitamin products, which are currently commercialized online by Amazon, a large and popular company focusing on electronic commerce in Western and Southern Europe. Many other companies, including among others iHerb^®^ and Alibaba.com, also sell dietary supplements online. Furthermore, it has been documented that the consumption of multivitamins is very common also in other geographical areas and populations (e.g., in Saudi Arabia among Arab populations [44,45]). Moreover, for obvious reasons inherent to its design, this labeling analysis based on internet marketplaces does not allow us to differentiate the contribution of societal attitudes, trade specificities, market characteristics, and relationships to environmental conditions in different areas and countries. Finally, supermarkets, grocery stores, and pharmacies also sell dietary supplements. Therefore, our analysis does not provide a whole picture of the over-the-counter commerce of multivitamins that contain vitamin D. Future studies are needed to address these issues. The current study, however, offers a useful knowledge basis and it might well anticipate the likely results of such studies, which we expect to be very similar. In addition, this study has at least three further relevant strengths. It is the first labeling study investigating the content of vitamin D in multivitamins available on Amazon. Second, several Amazon platforms were searched and compared. Finally, although the analysis was focused on vitamin D content, we also separately considered the content related to other vitamins.

Many authorities recommend a vitamin D supplementation in infancy and childhood [7,46]. On the other hand, the optimal dose of supplementation is still a matter of debate [47,48]. The present analysis did not survey the online shopping of vitamin D-containing multivitamin products aimed at these age groups. In England, only a few multivitamins from supermarkets or high street retailers supply the 400 IU daily dose advised by Public Health England for children over one year of age [49].

Cost and easiness are the major drivers of online shopping of multivitamin products. However, the online shopping of vitamin D-containing multivitamins is potentially not safe, mainly because missing the traditional relationship between practitioners (or pharmacists) and patients, consumers place themselves at some risk for side effects. Until appropriate safeguards have been implemented [16], patients and practitioners should be cautious. Nonetheless, physicians should not look at the practice of online shopping of multivitamins only as a threat, but rather as a potential opportunity for health promotion and a stimulus to improve communication with patients [50,51]. When advising vitamin D supplementation to a subject already on a multivitamin product, practitioners need to check that the multivitamin contains the appropriate amount of vitamin D. Finally, campaigns are needed to educate the public about the benefits and potential harms of online shopping of non-prescription multivitamin and multimineral supplements [50,51].

## 5. Conclusions

A dietary supplement is a product which contains ingredients intended to be added to the diet [3,4]. In addition to vitamins, common supplements include minerals, herbs, botanical compounds, amino acids, or live microbials. The main regulatory obligations are product safety and accuracy of the labeling that describes the product composition [14]. By law, dietary supplements are not drugs and, therefore, are not intended to prevent, treat, mitigate, or cure diseases [16]. The reasons individuals take a supplement vary, but critical motivations are to “improve” or “maintain” health. Women use supplements to foster bone health, men to improve cardiovascular “health or to lower blood fat levels” [52,53].

Given the extensive use of dietary supplements for health promotion, increased research efforts to address the safety and efficacy, and more investigation on the interplay of social and psychological determinants that inspire supplement choices are needed.

Finally, most dietary supplements are used by personal choice rather than upon physician recommendation [53]. In our opinion, the present labeling analysis points out that health care providers should play a critical role in counseling on dietary supplements. In particular, the choice of multivitamin preparations should accurately take into account their vitamin D content and individual modulators of vitamin D status such as sex, skin phenotype, seasonality, underlying chronic diseases, dietary habits, and sun exposure.

## Figures and Tables

**Table 1:** Characteristics of 199 multivitamin products containing vitamin D_3_, which are commercialized by Amazon marketplaces in Western and Southern Europe (amazon.es^®^, amazon.de^®^, amazon.it^®^, and amazon.fr^®^). Results are given as frequency or as median (with interquartile range).

	All	Spain	Germany	Italy	France
		amazon.es^®^	amazon.de^®^	amazon.it^®^	amazon.fr^®^
*n*	199	77	73	33	16
Vitamin D supply					
IU daily	250 (200–440)	200 (190–440)	336 (200–800)	600 # (200–1000)	250 (200–500)
<400 IU daily, *n*	108	49	37	13	9
400–800 IU daily, *n*	53	19	20	8	6
>800 UI daily, *n*	38	9	16	12	1
Number of vitamins *					
*n*	11 (10–12)	11 (10–12)	11 (10–12)	11 (10–12)	11 (10–12)
≤5 vitamins, *n*	20	11	3	4	2
6–10 vitamin, *n*	41	13	14	12	2
11–13 vitamins, *n*	138	53	56	17	12

# *p* < 0.05 versus Spain, Germany, and France; * other than vitamin D.

**Table 2:** Vitamin composition of 199 vitamin D_3_-containing multivitamins, which are commercialized by Amazon marketplaces in Western and Southern Europe (amazon.es^®^, amazon.de^®^, amazon.it^®^, and amazon.fr^®^).

	All	Spain	Germany	Italy	France
		amazon.es^®^	amazon.de^®^	amazon.it^®^	amazon.fr^®^
Products, *n*	199	77	73	33	16
Vitamin C	193	74	71	32	16
Vitamin E	183	70	69	29	15
Vitamin B_1_	181	67	71	29	14
Vitamin B_6_	181	68	69	29	15
Vitamin B_12_	180	68	70	27	15
Vitamin B_2_	177	67	68	29	13
Vitamin B_9_	173	66	65	27	15
Vitamin A	167	59	65	28	15
Vitamin B_3_	167	65	66	23	13
Vitamin B_5_	165	65	62	24	14
Vitamin B_8_	156	63	58	23	12
Vitamin K	90	26	39	19	6
Vitamin B_7_	13	0	5	6	2

## Data Availability

All data are given within the manuscript and Supplementary Materials.

## Supplementary Materials

The following supporting information can be downloaded at: https://www.mdpi.com/article/10.3390/nu15020326/s1, Table S1: Vitamin composition and daily vitamin D_3_ supply provided by 77 multivitamin supplements commercialized by amazon.es^®^; Table S2: Vitamin composition and daily vitamin D_3_ supply provided by 73 multivitamin supplements commercialized by amazon.de^®^; Table S3: Vitamin composition and daily vitamin D_3_ supply provided by 33 multivitamin supplements commercialized by amazon.it^®^; Table S4: Vitamin composition and daily vitamin D_3_ supply provided by 16 multivitamin supplements commercialized by amazon.fr^®^.
