# Supplementary material for: Vitamin D Supply of Multivitamins Commercialized Online by Amazon in Western and Southern Europe: A Labeling Analysis
Source: Nutrients. 2023 Jan 9;15(2):326. doi: 10.3390/nu15020326 (PMC9861848; doi:10.3390/nu15020326)
Supplement: Supplementary file 1 [file nutrients-15-00326-s001.zip › nutrients-2104972-supplementary.pdf]

Table S1. Vitamin composition and daily vitamin D_3 supply provided by 77 multivitamin supplements commercialized by amazon.es®.

	A	B_1	B_2	B_3	B_5	B_6	B_7	B_8	B_9	B_{12}	C	E	K	D_3 (units/day)
Vitamix liquid multivitamins		●	●	●		●		●	●	●				16
Natures Plus Source of Life Multivitamínico		●	●	●	●	●		●	●	●	●	●		65
Neo peques Gummies Vitazinc	●					●		●	●	●	●	●		75
Sotya Jalea Real con Vitaminas	●	●	●	●	●	●		●	●	●	●	●	●	90
Vitamix plus suplemento alimenticio	●	●	●	●	●	●		●	●	●	●	●		100
Solgar Female Múltiple		●	●	●	●	●		●	●	●	●	●	●	130
Fostprint plus Complemento Energético					●					●	●	●		185
Glory Feel Multivitaminas	●	●	●	●	●	●		●	●	●	●			200
Nature by Glory Feel Multivitaminas	●	●	●	●	●	●		●	●	●	●	●		200
Apisérum Vitalidad	●	●	●	●	●	●		●	●	●	●	●		200
Multicentrum efervescente para adulto		●	●	●	●	●		●	●	●	●	●	●	200
Vitamaze Multivitamin Daily	●	●	●	●	●	●		●	●	●	●	●	●	200
Multicentrum Complemento Alimenticio Multivitaminas con 13 Vitaminas y 11 Minerales	●	●	●	●	●	●		●	●	●	●	●	●	200
Pharmaton Mujer Vitaminas y minerales	●	●	●	●	●	●		●	●	●	●	●		200
Vitamaze Multivitamin + Mineralstoffe A–Z	●	●	●	●	●	●		●	●	●	●	●		200
Solaray Spectro Energy Multivitamin	●	●	●	●	●	●		●	●	●	●	●		200
Weider-Green Vitamin Complex	●	●	●	●	●	●		●	●	●	●	●	●	200
Solgar Nutrientes Prenatales	●	●	●	●	●	●		●	●	●	●	●		200
Apisérum Energia Vitamax	●	●	●	●	●	●		●	●	●	●	●		200
Sundt supplements liposomales multivitamin	●	●	●	●	●	●		●	●	●	●	●		200
MARNYS Cebida Multivitamínico	●	●		●		●					●			200
Sanct Bernhard A–Z càpsulas	●	●	●	●	●	●		●	●	●	●	●	●	200
Simplysupplements selenium with multivitamins and iron	●										●	●		200
SimplySupplements selenium with multivitamins and iron	●										●	●		200
Black Bee Pharmacy Jalea Multivitaminas	●	●	●	●	●	●		●	●	●	●	●		200
Leotrol energia vitaminas	●	●	●	●	●	●		●	●	●	●	●		200
Leotron vitalidad complex	●	●	●	●	●	●			●		●	●		200
Sundt supplements liposomales multivitamin	●	●	●	●	●	●		●	●	●	●	●		200
La santé par nature Vitamino immuné-fatigue	●	●	●	●	●	●		●	●	●	●	●		200
Fostprint sport Complemento Energético con Aminoácidos										●	●	●		200
Leotron energia mujer	●	●	●	●	●	●		●	●	●	●	●		200
Black Bee Jalea Real Inmuno Plus			●			●					●			200
Forte Pharma Energy Multivit Adulto	●	●	●	●	●	●		●	●	●	●	●		200
Victory Endurance All Day Energy	●	●	●	●	●	●		●	●	●	●	●		200
Forte Pharma Multivit Adulto	●	●	●	●	●	●		●	●	●	●	●		200
Simply supplements eye health multivitamin		●									●	●		200
Blissdefense multivitaminico para las defensas y sistema immunitario											●			200
Biotech USA Multivitamin for Men	●	●	●	●	●	●		●	●	●	●	●		200
PWD Vitalife Vitamins, minerals and antioxidants	●	●	●	●	●	●		●	●	●	●	●		200
Victory Endurance All Day Energy vitamins and minerals	●	●	●	●	●	●		●	●	●	●	●		200

Forte Pharma Multivit Adulto	•	•	•	•	•	•	•	•	•	•	•		200
Robis Multivitamínico con Vitaminas y Minerales	•	•	•	•	•	•	•	•	•	•	•		200
Prowise Multivitaminas y minerales	•	•	•	•	•	•	•	•	•	•	•	•	200
American supplement Multivitamin Combo	•	•	•	•	•	•	•	•	•	•	•		200
SimplyGo Immunity			•			•		•	•	•			200
Prisma Natural Hair 3.0		•	•	•	•	•	•	•	•		•		200
Ensure plus advance	•	•	•	•	•	•	•	•	•	•			230
Vitaworld Complejo Multivitamínico Prenatal *		•	•		•	•	•		•	•	•		250
Rilastil Sunlaude Oral—Suplemento Alimenticio				•							•		300
Solgar Fórmula Multivitamínica VM-75		•	•		•	•	•	•	•	•	•		400
Solgar Male Múltiple Complejo Multivitamínico y Multimineral para el Hombre		•	•	•	•	•	•	•	•	•	•		400
Lamberts one a day A to Z multivitamins and minerals	•	•	•	•	•	•	•	•	•	•	•	•	400
AMIX—Complejo Vitamínico - Multi Mega Stack con Vitaminas y Minerales	•	•	•	•	•	•	•	•	•	•	•	•	400
Amix multivitamins and minerals complex	•	•	•	•	•	•		•	•	•	•	•	400
Lamberts Multiguard	•	•	•	•	•	•	•	•	•	•	•	•	400
Pure Nutrition Daily Vitamins	•	•	•	•	•	•	•	•	•	•	•	•	400
Lamberts Multi Guard Iron Free	•	•	•	•	•	•	•	•	•	•	•	•	400
Meritene Fuerza y Vitalidad Fibra	•	•	•	•	•	•		•	•	•	•	•	440
FONTACTIV Protein Vital Complemento Nutricional	•	•	•	•	•	•	•	•	•	•	•	•	440
NOW Foods ADAM Multi-Vitamin for Men	•	•	•	•	•	•	•	•	•	•	•	•	500
Multicentrum Hombre 50+ ‡	•	•	•	•	•	•	•	•	•	•	•	•	600
Multicentrum Mujer 50+ ‡	•	•	•	•	•	•	•	•	•	•	•	•	600
CONCEIVE PLUS Vitaminas Fertilidad Femenina *		•	•	•	•		•	•	•	•	•		600
DonnaPlus Embarazo Complemento alimenticio para el embarazo *		•	•	•	•	•	•	•	•	•	•		600
Simplysupplements complete nutrition	•	•	•	•	•	•	•	•	•	•	•		600
Wellman conception *	•	•	•	•	•	•	•	•	•	•	•		600
Pregnacare conception *		•	•	•	•	•	•	•	•	•	•		600
Vegan Vitality Multivitamins & Minerals	•	•	•	•	•	•	•	•	•	•	•	•	800
Smarty Pants Women's Complete Multivitamin	•	•	•	•	•	•	•	•	•	•	•	•	1000
Centrum Silver Women 50+ ‡	•	•	•	•	•	•	•	•	•	•	•	•	1000
Simplysupplements Mature Man Multivitamin ‡	•	•	•	•	•	•	•	•	•	•	•	•	1000
One a day women's multivitamin/multimineral supplement	•	•	•	•	•	•	•	•	•	•	•	•	1000
Men's active sports multi	•	•	•	•	•	•	•	•	•	•	•	•	1000
Simplysupplements mature woman multivitamin ‡	•	•	•	•	•	•	•	•	•	•	•		1000
Vitalidad y defensas complex multivitamin	•	•	•	•	•	•	•	•	•	•	•		1000
New Chapter—Multivitamin prenatal perfecto *	•	•	•	•	•	•	•	•	•	•	•	•	1000
Alive immune with elderberry	•									•	•		1200

* recommended by the manufacturer for pregnancy and lactation; ‡ recommended by the manufacturer for the elderly.

Table S2. Vitamin composition and daily vitamin D_3 supply provided by 73 multivitamin supplements commercialized by amazon.de®.

	A	B_1	B_2	B_3	B_5	B_6	B_7	B_8	B_9	B_{12}	C	E	K	D_3 (units/day)
WARNKE heart vitamins multivitamin	●	●	●	●	●	●		●	●	●	●	●		67
WARNKE rich eye multivitamin		●	●	●	●	●		●	●	●	●	●		68
Fairvital Longevity multivitamin and multimineral		●	●	●	●	●		●	●	●	●	●		84
Zec+ All in One antioxidants & Vitamins	●	●	●	●	●	●	●			●	●	●	●	100
TNT Multivitamin Powder High dose Superfood Vitamin Complex	●	●	●	●	●	●		●	●	●	●	●		100
Vita2you Multi-Vitamin Caosules Vitamins & Minerals—Premium Quality	●	●	●	●	●	●		●	●	●	●	●	●	100
Seuren nutrient Super Multi Compleet	●	●	●	●	●	●	●		●	●	●	●		100
Pure encapsulations nutrient 950E capsules	●	●	●	●	●	●		●	●	●	●	●		134
EUNOVA Langzeit 50+ dietary supplement and multivitamin with minerals ‡	●	●	●	●	●	●		●	●	●	●	●	●	152
Vitamaze high-dose multivitamin capsules	●	●	●	●	●	●	●		●	●	●	●		200
Glory feel multivitamin	●	●	●	●	●	●		●	●	●	●	●		200
Doppelherz A–Z effervescent tablets orange passion flavour multivitamin supplement	●	●	●	●	●	●		●	●	●	●	●	●	200
CYB A–Z vitamins & minerals for men	●	●	●	●	●	●		●	●	●	●	●	●	200
Sanct Bernhard A–Z capsules multivitamin and mineral	●	●	●	●	●	●		●	●	●	●	●	●	200
CYB A–Z Vitamins & Minerals for Women	●	●	●	●	●	●		●	●	●	●	●	●	200
RENIGHT Multivitamin + Multimineral	●	●	●	●		●		●		●	●	●	●	200
Biotech USA Multivitamin	●	●	●	●	●	●		●	●	●	●	●		200
BEARS WITH BENEFITS Immunboost gummy bears	●					●			●	●	●	●		200
Multivitamin Vitamin Juice Liposomal	●	●	●	●	●	●		●	●	●	●	●		200
Fair vital daily multi vitamin complex	●	●	●	●	●	●		●	●	●	●	●	●	200
MED complete multi vitamin capsules	●	●	●	●	●	●		●	●	●	●	●	●	200
IronMaxx Multivitamin Capsules	●	●	●	●	●	●		●	●	●	●	●	●	200
My protein daily multi vitamin	●	●	●	●							●	●		200
A–Z generation 50+ multivitamin ‡	●	●	●	●	●	●		●	●	●	●	●	●	200
Hermes multivit	●	●	●	●	●	●		●	●	●	●	●	●	200
Doppelherz vegetariants vitamins + minerals		●	●			●				●				200
Lindens multivitamin A to Z	●	●	●	●		●			●	●	●	●		200
Frubiase Sport Orange Effervescent Tablets		●	●	●	●	●		●	●	●	●	●		200
Via vitamine multi vitamin and mineral A to Z	●	●	●	●	●	●		●	●	●	●	●	●	200
Gloryfeel high dose multivitamin		●	●	●	●			●	●	●	●	●		200
Nature by gloryfeel premium multivitamin extensive complex with bioactive forms	●	●	●	●	●	●		●	●	●	●	●		200
Fat2fit nutrition multivitamin A–Z	●	●	●	●	●	●		●	●	●	●	●	●	200
Medicus institute sinavita multivitamin capsules	●	●	●	●	●	●		●	●	●	●	●		200
Vita world natal multivitamin	●	●	●	●	●	●		●	●	●	●	●		250
Vitaworld multivitamin A–Z	●	●	●	●	●	●		●	●	●	●	●		250
Nutrilite double X amway Nahrungsergänzungsmittel	●	●	●	●	●	●		●	●	●	●	●		300
All stars multivitamin	●	●	●		●	●		●	●	●	●	●		336
Opti Men Nahrungsergänzungsmittel	●	●	●	●	●	●		●	●	●	●	●	●	400
Multivitamin energetic salus	●	●	●	●		●					●	●		400
BioTechUSA Multivitamin for Women	●	●	●	●	●	●		●	●	●	●	●		400
Heidelberger Chlorella Multivitamin active	●	●	●	●	●	●			●	●	●	●		400

Dietary supplement VM2000 multi vitamin Mineral	●	●	●	●	●	●		●	●	●	●	●		400
Solgar female multiple vitamin and mineral	●	●	●	●	●	●		●	●	●	●	●	●	400
Now foods adam men superior multi multivitamins	●	●	●	●	●	●		●	●	●	●	●	●	500
Optimum nutrition opti-women damen multivitamin	●	●	●	●	●	●		●	●	●	●	●	●	600
Pure Encapsulations All-In-One 50+ Multivitamin for more active ageing	●	●	●	●	●	●		●	●	●	●	●		600
Lemeda Multivitamin Man	●	●	●	●	●	●	●		●	●	●	●	●	600
Olimp vitamin		●	●			●			●	●	●			600
Vitasprint pro immune bottle – natural dietary supplement to activate defences						●					●			600
Jamieson multivitamin	●	●	●	●	●	●		●	●	●	●	●	●	600
ESN Vitamin Stack Multivitamin Supplements	●	●	●	●		●		●	●	●	●	●	●	800
Natural éléments premium multi multivitamin—comprehensive complex with bioactive forms and premium raw materials	●	●	●	●	●	●		●	●	●	●	●	●	800
Veganvitality vegan multivitamins and minerals with highly effective vitamins	●	●	●	●	●	●		●	●	●	●	●	●	800
Deva multivitamin and mineral supplement	●	●	●	●	●	●		●	●	●	●	●		800
Vincerol Plus Immun Kur Multivitamin	●	●	●	●	●	●	●			●	●	●	●	800
Health Germany femibion vitamine und minerakstöffe		●	●	●	●	●		●	●	●	●	●		800
Vital women multivitamin daily oral immune system booster supplement	●	●	●	●	●	●		●	●	●	●	●	●	800
Bayer one-a-day women's formula complete multivitamin	●	●		●	●	●		●	●	●	●	●	●	1000
Greenleaves vitamins—every day multivitamin	●	●	●	●	●	●		●	●	●	●	●	●	1000
Now foods eve superior multivitamin for women	●	●	●	●	●	●		●	●	●	●	●	●	1000
Multi complete multivitamin & mineral—Komplex Vegan	●	●								●			●	1000
Weider multivitamin complete A–Z premium complex with bioactive vitamins, trace elements and vegetable nutrients	●	●	●	●	●	●		●	●	●	●	●	●	1000
Nutri advances multi-essentials women's multivitamin + mineral complex	●	●	●	●	●	●		●	●	●	●	●	●	1000
Designs for health multivitamin	●	●	●	●	●	●		●	●	●	●	●	●	1000
Smarty pants women's complete multivitamin dietary supplement	●	●	●	●	●	●		●	●	●	●	●	●	1000
NOW foods adam multivitamin	●	●	●	●	●	●		●	●	●	●	●	●	1000
Simply supplements 50 plus formula ‡	●	●						●	●	●	●	●	●	1000
Nature's way alive premium formula multivitamin adult rubber animals	●	●	●	●	●	●		●	●	●	●	●		1200
Garden of Life Vegetarian Vitamin Supplement for Men Vitamin	●	●	●	●	●	●		●	●	●	●	●	●	1600
Olimp Vita-Min One	●	●	●	●	●	●		●	●	●	●	●		1600
Smarty Pants Women's Complete Multivitamin Dietary Supplement	●	●	●	●	●	●			●	●	●	●	●	2000
Scitec essentials daily vita-min	●	●	●	●	●	●		●	●	●	●	●		2000
Rainbow light unisex adult women one multivitamin	●	●	●	●	●	●		●	●	●	●	●	●	2000

‡ recommended by the manufacturer for the elderly.

Table S3. Vitamin composition and daily vitamin D_3 supply provided by 33 multivitamin supplements commercialized by amazon.it®.

	A	B_1	B_2	B_3	B_5	B_6	B_7	B_8	B_9	B_{12}	C	E	K	D_3 (units/day)
Epresat Multi-Vitam	●	●	●			●						●		180
ACT Vita Multivitamico	●	●	●	●	●	●		●	●	●	●	●	●	200
Multicentrum Adulti Effervescente	●	●	●		●	●		●	●	●	●	●	●	200
Nu3 premium vegan essential multivitaminico			●							●	●		●	200
Multivitaminico A–Z vit4ever	●	●	●	●	●	●	●		●		●	●		200
Leotron vitaminas jales	●	●	●	●	●	●		●	●	●	●	●		200
Vitamaze multivitaminico	●	●	●	●	●	●	●		●	●	●	●		200
Multivit integratore alimentare multivitaminico	●	●	●	●		●		●		●	●	●	●	200
Equilibra multivitamine & minerali	●	●	●		●	●		●	●	●	●	●		200
Multivitaminico masticabile nutrilite		●	●	●	●	●		●	●	●	●	●		200
Sustenium bioritmo 3 donna—integratore multivitaminico con antiossidanti e Sali minerali	●	●			●	●	●	●	●	●	●	●		200
Syform multivitaminico naturale	●	●	●	●		●		●	●	●	●	●	●	280
Syform multivitaminico naturale integratore alimentare	●	●	●	●		●		●	●	●	●	●	●	280
Vitabiotics Wellwoman Multivitaminico Gummies		●	●	●							●	●	●	400
GNC WOMEN'S Ultra mega active	●	●	●		●	●		●	●	●	●			400
Multicentrum uomo integratore	●	●	●	●	●	●		●	●	●	●	●	●	400
Dr. Giorgini Vitamine Maximum Complex	●	●	●		●	●		●	●	●	●	●	●	600
Jamieson Prenatal Multivitamin *	●	●	●	●	●	●		●	●	●	●	●	●	600
Dr. Giorgini integratore alimentare	●	●	●	●	●	●		●	●	●	●	●	●	600
Platinum naturals prenatal easymulti *	●	●	●	●	●	●			●	●	●	●	●	600
Platinum naturals easymulti teen for young men	●	●	●	●	●	●			●	●	●	●		800
Complete premium prenatal multivitamin	●	●		●	●	●		●		●	●	●	●	900
Multivitamine 12	●	●	●	●	●	●	●		●	●	●			960
Multivita Her	●	●	●	●	●	●	●	●	●	●	●	●	●	1000
Nutrivita multivitamines		●	●	●	●	●		●	●	●	●	●	●	1000
Il Multi nutrienti bioattivi	●	●	●	●	●	●	●	●	●	●	●	●	●	1000
Mutant Multi Athlete's vitamin	●	●	●	●	●			●	●	●	●	●	●	1000
Platinum naturals easymulti vegan	●	●	●	●		●		●	●		●	●		1000
Multivitamine vegetali e minerali ad alto contenuto di vitamine B_{12}, D_3, K_2	●	●	●	●	●	●		●	●	●	●	●	●	1000
Yamamoto research integratore di vitamine e minerali donna	●	●	●	●	●	●		●	●	●	●	●	●	1200
ExSeed Multi INtegratore di fertilità maschile	●					●			●		●			1400
Dr. Giorgini Idea C-difese immunitarie	●										●	●		2000
Women's multi			●		●	●		●	●	●	●	●		2000

* recommended by the manufacturer for pregnancy and lactation.

Table S4. Vitamin composition and daily vitamin D_3 supply provided by 16 multivitamin supplements commercialized by amazon.fr®.

	A	B_1	B_2	B_3	B_5	B_6	B_7	B_8	B_9	B_{12}	C	E	K	D_3 (units/day)
GPH diffusion multivitamines et minéraux	●	●	●	●	●	●		●	●	●	●	●		100
Multi vitamines & minéraux/NAKURU Boost	●	●	●	●	●	●		●	●	●	●	●		100
Puritae multivitamines et minéraux	●	●	●		●	●			●	●	●	●		134
Multivitamine liposomale en solution liquide pour un corps sain et résistant	●	●	●	●	●	●		●	●	●	●	●		200
Arkopharma azinc forme et vitalité	●	●	●	●	●	●		●	●	●	●	●		200
Tablets bargains multivitamines et fer	●	●	●	●	●	●	●		●	●	●	●	●	200
Trec nutrition multipack multivitamines et multimineraux	●	●	●	●	●	●	●	●	●	●	●	●		200
Multivit energy adult	●	●	●	●	●	●		●	●	●	●	●		200
Suravitasan multivitamines et minerals		●	●	●	●	●		●	●	●	●	●		300
Multicentrum hombre	●	●	●	●	●	●		●	●	●	●	●	●	400
12 defenses immunoenergy vitamines pour la fatigue	●					●			●	●	●			400
Now Foods Adam men superior multi-vitamine pour homme	●	●	●	●	●	●		●	●	●	●	●	●	500
Nutri advanced—immunoblast	●										●	●		500
Multicentrum complemento alimenticio mujer 50+ ‡	●	●	●	●	●	●		●	●	●	●	●	●	600
Vegan vitality multivitamines et minéraux végétaliens	●	●	●	●	●	●		●	●	●	●	●	●	800
Nature's way—alive multivitamines	●	●		●	●	●		●	●	●	●	●	●	1000

‡ recommended by the manufacturer for the elderly.
